# A Workshop for Training of Basic Neurosurgical Skills "From Microsurgery to Endoscopy": A Stepping Stone for Young Neurosurgeons

**DOI:** 10.7759/cureus.3658

**Published:** 2018-11-30

**Authors:** Nikolay Lasunin, Denis A Golbin

**Affiliations:** 1 Neurosurgery, Nikolay Nilovich Burdenko National Medical Research Center for Neurosurgery, Moscow, RUS

**Keywords:** microsurgery, endoscopy, microneurosurgery, practical training, neurosurgery, neuroendoscopy, surgical simulation

## Abstract

Background

The aim of the project was to show the peculiarities of manipulations in two-dimensional (2D) visualization and to enlighten young neurosurgical residents for further independent training of their manual skills in the anatomic lab. The course followed a step-by-step training program starting with artificial models in a static 2D exoscopic view field with subsequent transition to cadaveric animal models and the use of dynamic four-hand techniques and endoscopy.

Materials and methods

Since 2015, two 2-day workshops and four 1-day workshops have been organized. All courses consisted of short theoretical and prevailing hands-on practical part and were designed mainly for manual skills training. For optimal practical training, each pair of trainees were engaged with a separate working place equipped with a video system, exoscope, drill, suction, and a set of microsurgical and endoscopic instruments. A total of 48 trainees, including residents of the Nikolay Nilovich (NN) Burdenko National Medical Research Center of Neurosurgery (NMRCN), the Russian Medical Academy of Continuous Postgraduate Education, and other institutions from different regions of Russia, completed the program. Analysis of evaluation sheets revealed that over 90% of trainees were under the age of 30.

Results

The key idea – accommodation to endoscopy through a series of microsurgical exercises in 2D visualization – has been successfully actualized and met with interest. After analyzing the questionnaires, we found that an overall satisfaction rate was high. The vast majority of trainees intended to gain further experience and apply new techniques in their clinical neurosurgical and microsurgical practice. The number of practiced techniques and the quality of the provided equipment were considered by the participants as good or very good. The highly individualized training course with a participant/tutor ratio of 4:1 and the use of tissue models (no sacrifice of living animals) was well appreciated.

Conclusion

The demand for a workshop indicates a lack of such training activities for young professionals, such as the one we presented herein. Evaluation of the courses by the trainees showed that the contents of workshops corresponded to their tasks and expectations, regardless of their previous experience. The workshop was not only the 'stepping stone' from which the path of practical self-development should begin but also initiated the development of a whole series of specially focused training workshops on microsurgery and endoscopy for neurosurgeons.

## Introduction

Today, an increase in the number of residents choosing neurosurgery as their future specialty is being observed in the Russian Federation [1]. At the same time, neurosurgery combines a huge amount of different pathologies and treatment modalities while the price of the surgeon’s mistake is extremely high in terms of the low reparative capacity of the central nervous system. However, an opportunity to learn and improve practical skills in microneurosurgery is usually restricted to clinical settings on patients undergoing operations. Combined with a large amount of theory, it makes long-term training in neurosurgery essential. Training of manual skills of young neurosurgeons is a critical challenge during residency and further surgical practice. Given the presence of an anatomic lab in the majority of clinics, the idea of open master classes to initiate practical training was introduced. The initial experience in microsurgical and endoscopic techniques under the supervision of skilled surgeons (both authors) was undertaken at the Nikolay Nilovich (NN) Burdenko National Medical Research Center for Neurosurgery (Moscow, Russia). The aim of the project was to show the peculiarities of manipulations in two-dimensional (2D) visualization and to enlighten young residents for the further independent training of their manual skills in the anatomic lab.

The key idea of our workshop was to provide an initial experience with microsurgical tools and endoscopic dynamic view so that the trainees could learn and practice on tissue preparation and microvascular and nerve anastomoses using 2D visualization. Trainees were selected on the basis of their initial skills. They were supposed to be already familiar with microsurgical technique and instrumentation and basic skills of microsurgical suturing on models, including end-to-end artery and nerve anastomosis using a neurosurgical microscope.

The principal endpoint of the workshops was to familiarize the trainees with the basic skills of endoscopic surgery; however, the transition to endoscopy required bimanual microsurgical manipulations in the 2D view. Thus, each workshop consisted of two blocks: microsurgical and endoscopic. Though courses were focused on practical training, lectures were given by the instructors at the beginning of each block. The first lecture represented a review of microsurgical technique (basic microsurgical instruments, sutures, microvascular anastomosis, nerve anastomosis). A short briefing following that was intended to familiarize the trainees with the equipment and instrumentation and emphasize all handling and safety procedures. The other lecture was in the second part and discussed philosophy, advantages, and limitations of endoscopic techniques in neurosurgery.

Our course followed a step-by-step training program starting with artificial models in a static 2D exoscopic view field with a subsequent transition to cadaveric animal models and the use of dynamic four-hand techniques.

## Materials and methods

Arrangement and participants

**Figure 1:**
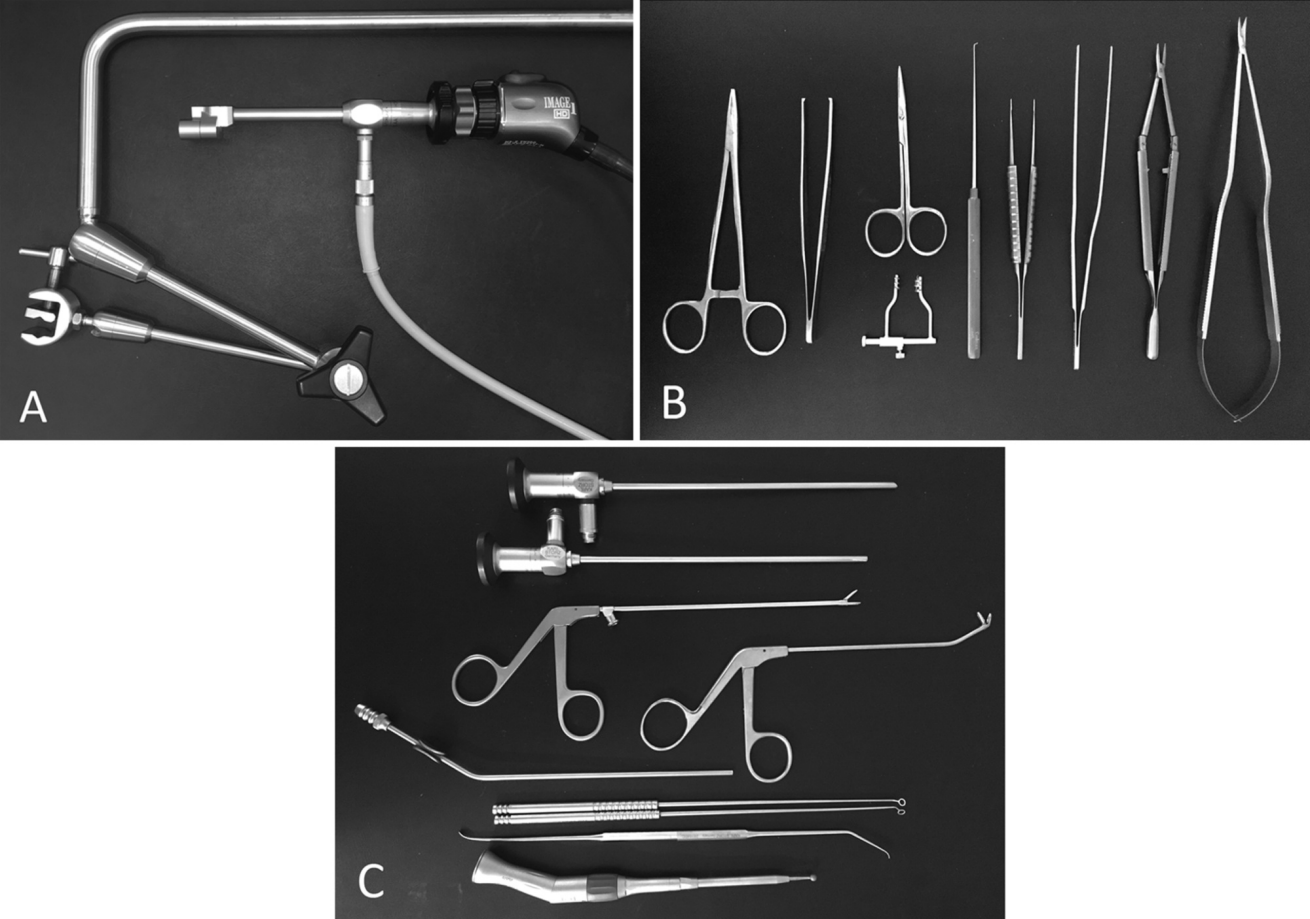
Instrumentation used for training A: exoscope and holding arm. B: instruments for microsurgical part (needle holder, forceps, scissors, retractor, hook dissector, microforceps, microneedle holder, microscissors). C: instruments used for endoscopic part (0- and 45-degree 4 mm rigid telescopes, straight and curved Blakesley forceps, suction cannula, curettes, dissector, electric drill).

The practical training course “Basic Microsurgical and Endoscopic Training in Neurosurgery” was conducted at the NN Burdenko National Medical Research Center for Neurosurgery (Moscow, Russia; further mentioned as NN Burdenko NMRCN) especially for neurosurgical residents. This course did not require institutional approval. Since 2015, two 2-day workshops and four 1-day workshops have been organized. All courses consisted of a short theoretical and prevailing hands-on practical component and were designed mainly for manual skills training. For optimal practical training, each pair of trainees was set up with a separate working place equipped with a video system, exoscope, drill, suction, and a set of microsurgical and endoscopic instruments (Figure 1).

**Figure 2:**
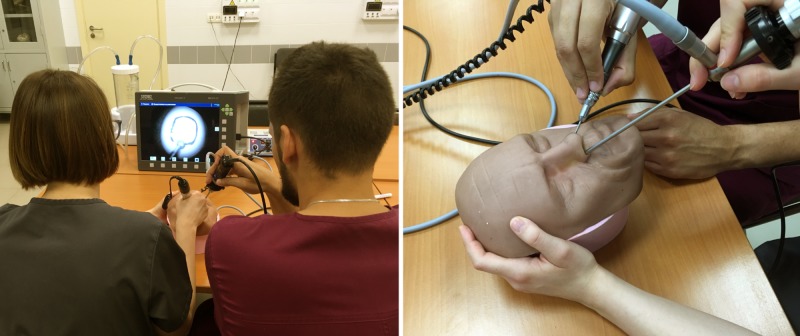
Supervision of a couple of the trainees during endoscopic manipulations Left: overall view of the working place. Right: bi-nostril positioning of the instruments (both trainees are involved in the manual exercise).

Each course was designed for four working places (eight residents) to ensure optimal supervision and comfort, and the tutor: trainee ration was not less than 1:4. The tutors recruited from the neurosurgical department of NN Burdenko NMRCN had sufficient experience in microneurosurgery, endoscopic techniques, and in experimental microsurgical models. All endoscopic video systems were in the supervisors’ field of view, which provided close communication and immediate feedback and assistance (Figure 2).

A total of 48 trainees, including residents from NN Burdenko NMRCN, the Russian Medical Academy of Continuous Postgraduate Education, and other institutions from different regions of Russia, completed the program. Analysis of the evaluation sheets revealed that over 90% of trainees were under the age of 30. Eight (16.7%) of the participants were pregraduate medical students. More details regarding trainees are listed in Table 1.

**Table 1:** Distribution of the trainees according to the specialties and level of expertise ENT: Ear, Nose, and Throat

Trainees		Number	%
Students		8	16.7%
Residents		37	77.1%
	Neurosurgery	34	
	ENT	2	
	Other	1	
Physicians (neurosurgery)		3	
Total		48	6.3%
Specialties		Number	%^*^
Neurosurgery		37	92.5%
ENT		2	5%
Other		1	2.5%

Hardware

The exoscopes, endoscopes, and microsurgical tools were provided by the Neuroanatomy Laboratory of NN Burdenko NMRCN and the Moscow Office of Karl Storz GmbH and Co. KG, which guaranteed high quality and a wide range of dedicated instruments and equipment.

Microsurgical training

The first block started with dura suturing simulation on the model made of two latex gloves one inside the other. The inner glove was filled with water, and the outer one was incised in order to simulate safe suturing over the water balloon representing the arachnoid membrane over the brain. This simulation was accomplished using a 2D view of the video system.

To practice microvascular anastomosis, we used perfused fresh chicken legs purchased in a supermarket. To provide a simulation of arterial 'blood' flow during microvascular anastomosis suture, we used a water pump, red dye, and a beeper indicating heart rate (Video 1).

**Video 1:** Exercise of microvascular anastomosis by trainees The two-dimensional visualization is obtained using the exoscope and video system, and the loss of depth creates an additional challenge for the trainees, making familiar manipulations more difficult.

End-to-end anastomosis, as well as arteriovenous shunts, could be practiced with this model after dissection of the femoral bundle. The interrupted suture technique with a rotation of the vessel was practiced first. With the improvement of skills in controlling the microsurgical instruments, suture material, and tissue, the continuous suture technique was introduced and tried by the participants, if sufficient time was left.

The tissue model for nerve anastomosis was the sciatic chicken nerve. On the same specimens, the sciatic nerves were cut and re-anastomosed using epineural sutures. Both these models provided four-hand surgery training of extended surgical skills in 2D visualization, which is significantly more difficult than with a surgical microscope; however, these conditions help to accommodate to endoscopic view.

Endoscopic training

The second section included 0- and 45-degree endoscopic visualization, two- and four-hand endoscopic manipulations, using a high-speed drill, and a simulation of an endoscopic transnasal tumor removal.

For the very basic purposes, a red pepper model was used. First, seed removal under the control of zero-degree telescopes was practiced. As the surgical skill grew, the participants proceeded to the 45-degree telescopes, and finally, to the three- and four-hand technique.

To train precise manipulations under endoscopic control in a narrow space, we used a rubber model of the face and paranasal sinus and chicken eggs [2]. The next step was practicing the drilling of the shell of raw eggs under the endoscopic control (Video 2). The goal was to remove the shell on the area of 1 square cm without any damage to the underlying tissues using a diamond burr. In the final stage exercise, semi-boiled eggs were used. Placed in the area of the sphenoid sinus, they simulated a soft skull base tumor (e.g., pituitary adenoma). After the wider shell removal, participants cut through the egg white and removed the yolk in the four-hand technique using suction and curette (Video 3).

**Video 2:** Drilling of the eggshell

**Video 3:** Simulation of cystic/solid tumor removal using an egg model

## Results

The key idea – accommodation to endoscopy through a series of microsurgical exercises in 2D visualization – has been successfully actualized and met with interest. After two 2-day courses, our impression and feedback helped us to come to the decision of a reduction of the course to a single day. Albeit more intensive, the program became more convenient both for organizers and for trainees. This may be of special importance to a participant who arrives from a distant region. Another key point was stimulating everyone to be focused and organized in order to keep to the time limit. 

The timing of the workshop allowed for effective rest during two coffee breaks lasting 15 minutes each and a long lunch break (60 minutes). The latter was used to separate the two parts of the workshop and to rearrange workplaces while the trainees were away. The overall length of the workshops was six hours of pure working time, plus a total of 90 minutes of breaks.

As expected, younger participants succeeded better in getting accustomed to the 2D endoscopic visualization as compared to the experienced physicians. However, at the same time, young residents were more prone to make mistakes in handling the endoscope. The most common mistakes, according to our observations, included tilt and rotation of the camera head, uncomfortable posture, and intercrossing of the telescope and instruments, leading to a failure to visualize an instrument in the field. It was indicative that, due to the high level of flexibility in learning, the young trainees succeeded in teamwork during the tasks requiring three- or four-hand manipulations.

Assessment of comfort during the training process was shadowed by an excessive number of participants during one of the workshops (nine instead of eight). Unfortunately, one of four workplaces was overcrowded, and during all exercises, one of three trainees was forced to be out of the process.

Despite the fact that we did not perform any scoring scale for the quality or speed of tasks evaluation, in order to assess and adapt the course concept, all participants were encouraged to fill in an evaluation sheet with a Likert-type scale at the end of the course.

**Figure 3:**
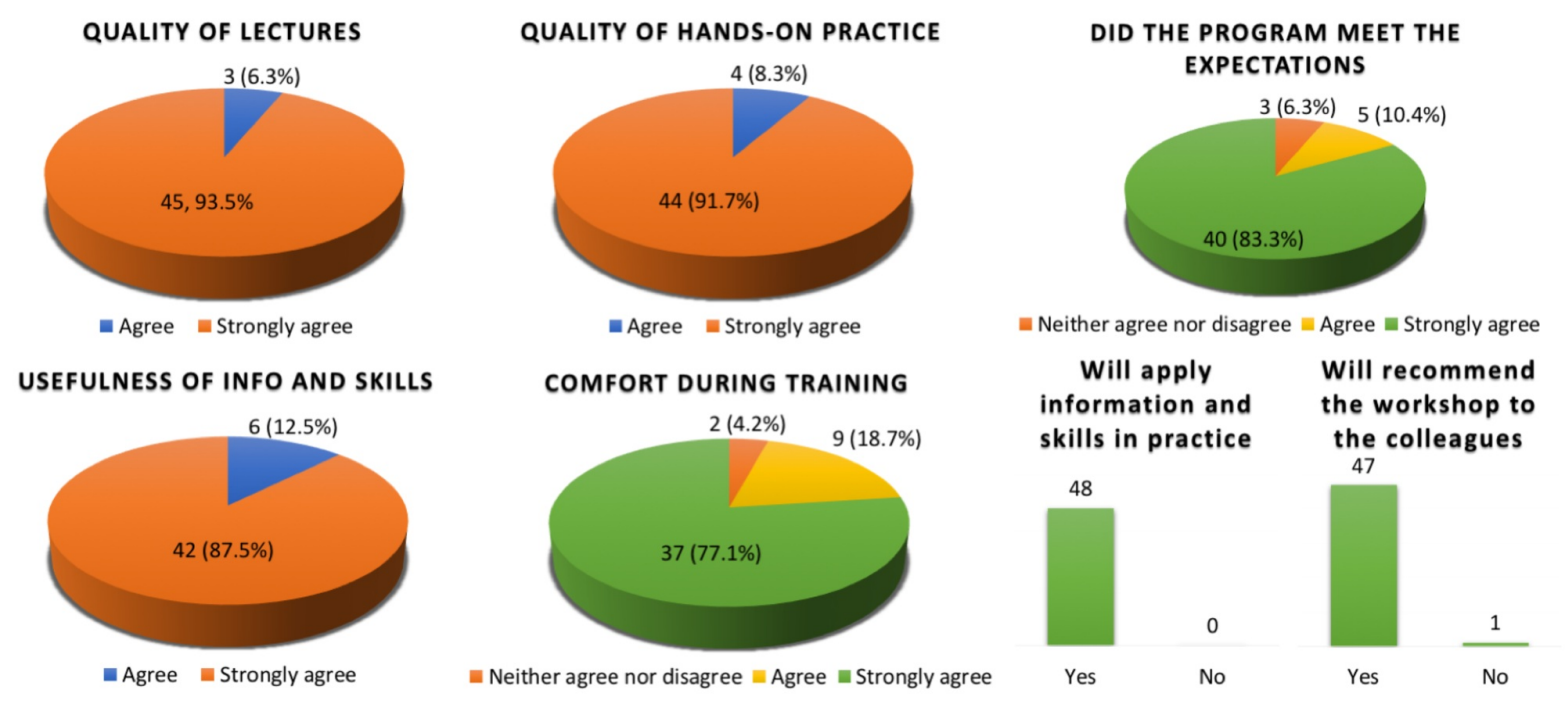
Results of the evaluations of the workshops by participants obtained after analysis of feedback forms

After analyzing the questionnaires, we found that an overall satisfaction rate was high (Figure 3). The vast majority of trainees intended to gain further experience and apply new techniques in their clinical neurosurgical and microsurgical practice. The number of practiced techniques and the quality of the provided equipment were considered by the participants as good or very good. The proportion of the lectures and practical training was considered reasonable. The highly individualized training course with a participant/tutor ratio of 4:1 and the use of tissue models (no sacrifice of living animals) were well appreciated. Additionally, we offered (for interested participants) some opportunities, such as additional time for practice, skull-base dissection on non-living animal models, work on living animal models, and attendance on clinical rounds.

## Discussion

Microsurgical techniques are widely used in almost all fields of surgery, such as thoracoabdominal surgery, urology, gynecology, plastic surgery, vascular surgery, etc. [3-6].⁠ 

However, throughout the 20th century - the era of the formation and heyday of neurosurgery – microsurgical training in neurosurgery started on tissue models, living models, or in the operating room [7-8] and was like a field test of all theoretical knowledge so it could not be called “training” at all. However, since the end of the 20th century, due to changes in regulations in many countries (for ethical and religious reasons), a serious lack of anatomical material resulted, which led to the active development of various simulation models.

Generally, these models can be divided into three main groups: biological [2, 9-12], artificial [13-14], and virtual [15-18]. The main advantage of biological models (living animals or tissues) is the possibility of achieving maximum similarity with the real intraoperative pattern. However, these models have serious drawbacks: first of all, it is the complexity of use (the need for vivarium, animal delivery system, disposal of biological waste), as well as the high price of "consumables". The advantage of artificial models is the low price. While these models help to familiarise the trainees with microsurgical instruments, equipment, and techniques, they definitely lack the realism of biological models. Virtual models have received the most active development during the last 10 - 15 years when technologies appeared both for creating high-quality virtual models and for the technical component with feedback (like three-dimensional (3D) touch).

Neuroendoscopy is becoming an increasingly common area for neurosurgical techniques, and it is long overdue for young neurosurgeons to be given the opportunity to practice "from scratch". Work in a narrow corridor with a 2D image requires fundamentally different skills.

Obtaining non-microsurgical skills is also a crucial aspect of the course. Fourteen percent of our participants have no intention of transferring the acquired skills directly into clinical practice; however, the experience of delicate dissection, tissue manipulation, manipulations in the 2D field of view, and magnified optical control also have a great impact in refreshing macroscopic surgical skills.

It is noteworthy that the master class aroused interest not only among the residents but also neurosurgeons with experience.

The literature describes a large number of specialized or universal training courses on microsurgery [7, 12-14, 19-20]⁠, the individual models for working out highly specialized skills [21], the various systems for assessing the learner, and the effectiveness of training [12-13, 22]. The disadvantage of most such courses is the narrow specialization, the high cost (due to the high price of models or consumables) for narrowly-specified courses, and a long duration (correspondingly high time costs of cadets and teachers) and high costs (employment of teachers and premises).

We present the training course concept that could be carried out in almost any surgical department. The organization requires only portable exoscope systems and a minimal set of microsurgical instruments that could be obtained (or rented) through cooperation with local distributors of eligible companies.

## Conclusions

The demand for a master class indicates a lack of similar training activities for young professionals. The questioning of cadets showed that the contents of the master class corresponded to the tasks and expectations of the trainees, regardless of their work experience. The master class was not only the first step from which the path of practical self-development should begin but also initiated the development of a whole series of thematic training courses on microsurgery and endoscopy for neurosurgeons.
